# Optimization of Protein Extraction Method for 2DE Proteomics of Goat’s Milk

**DOI:** 10.3390/molecules25112625

**Published:** 2020-06-05

**Authors:** Muzammeer Mansor, Jameel R. Al-Obaidi, Nurain Nadiah Jaafar, Intan Hakimah Ismail, Atiqah Farah Zakaria, Mohd Azri Zainal Abidin, Jinap Selamat, Son Radu, Nuzul Noorahya Jambari

**Affiliations:** 1 Laboratory of Food Safety and Food Integrity, Institute of Tropical Agriculture and Food Security, Universti Putra Malaysia, Serdang 43400 UPM, Selangor, Malaysia; muzammeer.mansor@gmail.com (M.M.); nadyajaafar94.nj@gmail.com (N.N.J.); jinap@upm.edu.my (J.S.); son@upm.edu.my (S.R.); 2 Department of Biology, Faculty of Science and Mathematics, Universiti Pendidikan Sultan Idris, Tanjong Malim 35900, Perak, Malaysia; 3 Department of Paediatrics, Faculty of Medicine and Health Sciences, Universiti Putra Malaysia, Serdang 43400 UPM, Selangor, Malaysia; intanhakimah@upm.edu.my (I.H.I.); mazrizb@upm.edu.my (M.A.Z.A.); 4 Department of Otorhinolaryngology, Faculty of Medicine and Health Sciences, Universiti Putra Malaysia, Serdang 43400 UPM, Selangor, Malaysia; atiqah@upm.edu.my; 5 Department of Food Science, Faculty of Food Science and Technology, Universiti Putra Malaysia, Serdang 43400 UPM, Selangor, Malaysia

**Keywords:** 2DE, gel electrophoresis, goat’s milk, protein extraction, proteomics

## Abstract

Two-dimensional electrophoretic (2DE)-based proteomics remains a powerful tool for allergenomic analysis of goat’s milk but requires effective extraction of proteins to accurately profile the overall causative allergens. However, there are several current issues with goat’s milk allergenomic analysis, and among these are the absence of established standardized extraction method for goat’s milk proteomes and the complexity of goat’s milk matrix that may hamper the efficacy of protein extraction. This study aimed to evaluate the efficacies of three different protein extraction methods, qualitatively and quantitatively, for the 2DE-proteomics, using milk from two commercial dairy goats in Malaysia, Saanen, and Jamnapari. Goat’s milk samples from both breeds were extracted by using three different methods: a milk dilution in urea/thiourea based buffer (Method A), a triphasic separation protocol in methanol/chloroform solution (Method B), and a dilution in sulfite-based buffer (Method C). The efficacies of the extraction methods were assessed further by performing the protein concentration assay and 1D and 2D SDS-PAGE profiling, as well as identifying proteins by MALDI-TOF/TOF MS/MS. The results showed that method A recovered the highest amount of proteins (72.68% for Saanen and 71.25% for Jamnapari) and produced the highest number of protein spots (199 ± 16.1 and 267 ± 10.6 total spots for Saanen and Jamnapari, respectively) with superior gel resolution and minimal streaking. Six milk protein spots from both breeds were identified based on the positive peptide mass fingerprinting matches with ruminant milk proteins from public databases, using the Mascot software. These results attest to the fitness of the optimized protein extraction protocol, method A, for 2DE proteomic and future allergenomic analysis of the goat’s milk.

## 1. Introduction

Cow’s milk protein allergy (CMPA) is prevalent among young children and has been reported to affect around 2.5% of children during their early years [1,2]. Based on studies conducted on a large population of CMPA patients, the major allergens are caseins (CN), α-lactalbumin (ALA), and lactoglobulin (β-LG), which are the most abundant proteins in cow’s milk, yet low-abundance proteins such as bovine serum albumin (BSA), lactoferrin (LF), and Immunoglobulin (Ig) had also been suggested to be able to induce allergic sensitization [3]. Traditionally, goat’s milk has been proposed as a hypoallergenic cow’s milk substitute for CMPA patients. Despite their close milk protein homology due to sharing the same phylogenetic origin [4], about 40% of cow’s milk protein allergic patients were estimated to have tolerance toward goat’s milk proteins [5]. Genetic polymorphisms observed in different goat breeds had been indicated to affect the milk casein fractions, including αs1-casein (αs1-CN) [6,7] and kappa-caseins (κ-CN) [8]; thus, they potentially affect their allergenicity. Most of the previous studies performed on evaluating the goat’s milk protein cross-reactivities with cow’s milk allergens [9,10] employed 1D SDS-PAGE and IgE-immunoblotting to suggest presumptive allergenic proteins, yet there has been no study focusing on profiling the complete allergenic proteins (allergenome) of milk from different goat breeds and their cross-reactivities with cow’s milk allergens.

The allergenome of milk is highly complex due to the following: (1) the effects of genetic polymorphisms of the individual and specific breeds of ruminants, as observed in the presence of different alpha caseins (α-CN), beta caseins (β-CN), κ-CN, ALA, and β-LG variants in different cow’s breeds [11,12,13,14]; and (2) post-translational modifications, including glycosylation, phosphorylation, acetylation, and proteolytic cleavage [14]. Changes in amino acid sequence, deletion of peptide fragments, and post-translational modifications due to genetic polymorphisms greatly affect the milk proteins’ functional properties, such as the stability of micelles, solubility, and digestibility [15]. These factors contributed to the changes of the milk protein structures and functions, leading to potential changes in the allergenicity of the proteins.

Two-dimensional electrophoretic (2DE)-based proteomics is a combination of protein separation by isoelectric focusing and SDS-PAGE and mass spectrometric identification. It is an indispensable top-down approach for resolving and identifying multiple protein isoforms, in addition to providing visual confirmation in protein differences and changes between samples [16]. Due to these attributes, 2D-based proteomic method is adopted as a standard approach in food allergenomics for the identification and characterization of novel food protein allergens. For the allergenomic approach, the separated protein spots on 2DE are transferred onto nitrocellulose membrane, probed with serum IgE from allergic patients, and the IgE-bound proteins are identified by mass spectrometry [17]. This approach had been previously employed for the identification of cow’s milk allergenic protein isoforms [18], novel rice allergens [19], and other allergens from animal and plant origins, as extensively reviewed by Di Girolamo et al. [17]. Moreover, 2DE proteomics has also been used to investigate allergen variability in soybean seeds from GM and non-GM crops [20] and to detect masked cow’s milk allergen, αs1-CN, in human colostrum [21].

However, the most critical step to achieve accurate, reliable, and reproducible results in the 2DE-based proteomic study is the sample preparation [22]. An ideal sample preparation should be able to solubilize and extract all proteins from the sample sources, without any structural or chemical modification, eliminate any interfering compounds, and keep the sample in a compatible form with mass spectrometric analytical methods [23,24]. Effective sample preparation for proteomics requires the disruption of the chemical bonds, which include the hydrogen bond, disulfide bond, hydrophobic interactions, van der Waals force, charge–dipole bond, and electrostatic interactions that hold the protein structure together in its native state, and dissociation of protein interaction and association with other proteins, biomolecules, and matrices [25]. This can be achieved by combining different additives such as salts, detergents, chaotropes, reducing agents, and ampholytes in the solubilizing and extraction buffers [25]. Ineffective extraction methods may result in smearing, horizontal and vertical streaking, and protein loss due to poorly resolved protein spots on the 2DE gel.

Although several goat’s milk proteome studies have been performed for different purposes [15,26,27], our study is the first that attempts to establish an optimized extraction method suited for the 2DE-based proteomic analysis of whole goat’s milk proteins. The protein structures of goat’s milk are known to be similar to cow’s milk, where its solid phase is composed of caseins that are bound together into micelles by calcium phosphate and traces of citrate, potassium, sodium, and magnesium, as well as a soluble phase that is made up of whey proteins [28]. However, differences in the composition of caseins and higher mineralization of goat’s milk compared to cow’s milk cause the goat casein micelles to be smaller, less hydrated, and more susceptible to high temperature, as well as showcase higher loss of β-CN than cow’s milk micelles [28]. Thus, the differences in goat’s milk matrices compared to cow’s milk may cause incompatibility of optimized extraction methods that have been established for cow’s milk [29] to our 2DE-based proteomic approach in analyzing goat’s milk proteomes.

The present study aims to evaluate and compare the efficacies of three different protein extraction methods based on the 2DE maps and matrix-assisted laser-desorption ionization-time-of-flight tandem mass spectrometric (MALDI-TOF/TOF MS/MS) analysis, using milk from Saanen and Jamnapari goats. Here we adapted and modified extraction method that has been used in studying milk proteins and allergens [30,31]. We chose Saanen and Jamnapari goats in this study, as they are among the most commonly bred dairy goats in Malaysia due to their high milk production [32]. Although the chemical compositions of both breeds have been rigorously studied and compared [33,34,35], differences in their protein isoforms, including allergenic contents, remain to be investigated.

## 2. Results

### 2.1. Protein Concentration Determination of Milk Extracts from Different Extraction Methods

**Figure 1:**
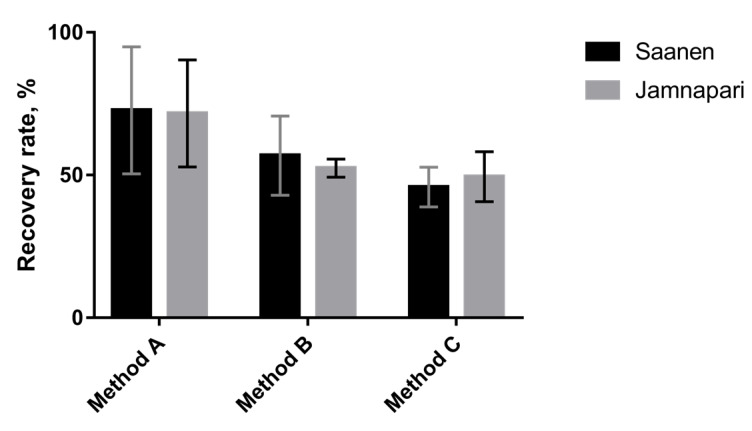
The protein recovery rate of each extraction method is presented as a percentage of extracted protein concentration calculated against skim milk protein concentration. Error bars are from Bradford assay technical replicates and presented as SD (*n* = 3).

Protein extraction efficiency and reproducibility were evaluated based on the amount of extracted protein from 0.5 mL of skim goat’s milk samples. Protein concentration of each extract was tabulated in Table 1. Extraction using method A, which is a modified urea-based extraction method by dilution with a different combination of 3-[(3-cholamidopropyl) dimethylammonio]- 1-propanesulfonate (CHAPS), urea, thiourea, ampholytes, and dithiothreitol (DTT) recovered the largest amount of proteins, with results of 120.54 ± 22.26 and 134.62 ± 18.76 mg/mL from Saneen and Jamnapari goat’s milk, and the highest recovery rates for skim milk at 72.68% and 71.65% for respective goat breeds (Figure 1). This is followed by method B, which is a methanol/chloroform-based protein triphasic separation method, and method C, a modified sodium-sulfite-based method that consists of Tris/HCl, Tween 20, sodium dodecyl sulphate (SDS), and sodium sulfite.

### 2.2. Evaluation of SDS-PAGE Band Profiles of Saanen and Jamnapari Goat’s Milk Protein Extracts from Different Extraction Methods

**Figure 2:**
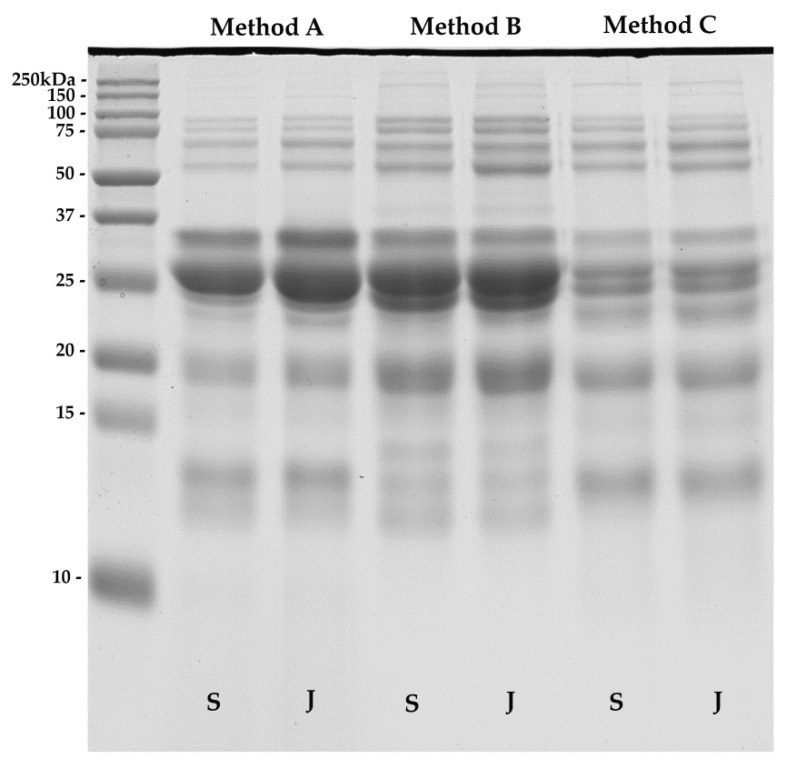
SDS-PAGE profiles of goat’s milk from Saanen (S) and Jamnapari (J) extracted using three different extraction methods: (A) urea/thiourea-based extraction, (B) methanol/chloroform-based triphasic separation, and (C) sodium sulfite-based extraction.

The three protein extraction methods compared in this study were assessed by using 1D SDS-PAGE, as shown in Figure 2. The intensity of resolved band patterns on SDS-PAGE gave initial information on the quality and quantity of the extracted proteins. An equal amount of proteins, 20 µg, was loaded from each extract. Proteins were separated between 10 and 250 kDa in 12% polyacrylamide gel. The electrophoretic profiles of each protein extract were similar, but method A and B produced more-intense bands, particularly on 25 to 37 kDa area, compared to method C. While SDS-PAGE profiles from methods A and C exhibited more intense bands around 10 to 15 kDa area than method B, method B recovered more protein bands within this region. In terms of the quality of the proteins, all three extraction methods showed good band separation of proteins, indicating that the proteins remained intact and not denatured.

### 2.3. Comparison of the Protein Profiles between Different Goat’s Milk Extracts, Using 2DE Gels

**Figure 3:**
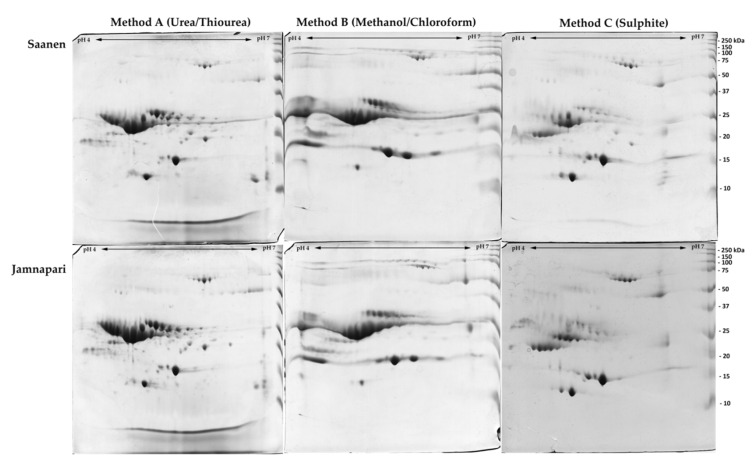
Two-dimensional electrophoretic (2DE) profiles of three different total protein extraction methods: (A) urea/thiourea-based, (B) methanol/chloroform triphasic separation, and (C) sodium sulfite-based extraction methods, of milk from (S) Saanen and (J) Jamnapari goats. Protein extracts (400 µg) were run on 13 cm pH 4–7 IPG strip and resolved on 12% bis-acrylamide gel.

Extracted milk proteins from both breeds, using three different extraction methods, were further separated on the 2DE gel, based on their isoelectric point and molecular weight. Images of 2DE gels of the protein extracts of Saanen and Jamnapari goat’s milk after Coomassie brilliant blue (CBB) staining revealed that method A had resolved protein spots with the best resolution, the least amount of horizontal and vertical streaking, and clearer visible elliptical protein spots than methods B and C (Figure 3). The intensity of protein spots that were closely clustered together in the pI region of pH 4–5 and molecular weight of around 20 to 37 kDa was the highest in samples of method A, followed by methods B and C. Method B showed the poorest resolution of 2DE gels, with the most horizontal streaking. While method C may not have a critical amount of streaking and had good protein spot resolution, the resolved protein spots were less intense compared to the other methods, specifically for proteins in the region of pH 4 to 5 and molecular weight of around 20 to 30 kDa. Gel image analysis was then performed, using Progenesis SameSpot software to quantify protein spots, as summarized in Table 2. Method A was shown to resolve the highest total number of protein spots for both breeds: 199 ± 16.1 for Saanen, and 267 ± 10.6 for Jamnapari, followed by method B and method C.

### 2.4. Protein Identification Via MALDI-TOF/TOF MS/MS

**Figure 4:**
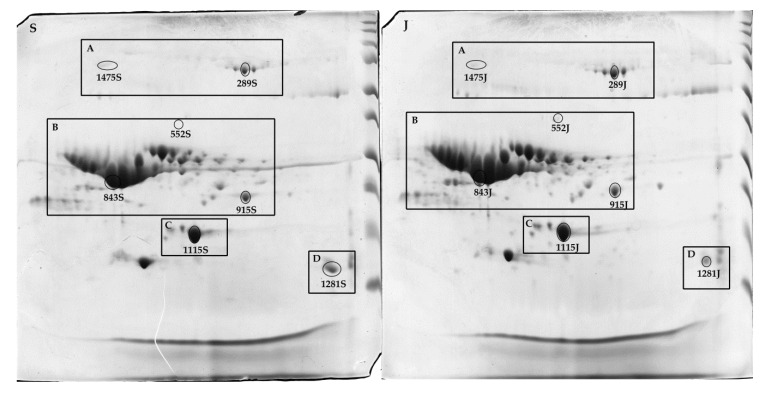
Spots chosen for protein identification by MALDI-TOF/TOF MS/MS for (S) Saanen and (J) Jamnapari: (A) serum albumin region [36,41]; (B) casein region [37,38]; (C) beta-lactoglobulin region [26,39], and (D) S100 calcium-binding protein region [40].

As method A produced gels with the best spot resolution and the highest total number of protein spots, method A was chosen as the method of choice. To confirm that, six spots from each breed gel extracted from method A were randomly selected from specific milk protein regions of proteins that were identified in previous studies as serum albumin, CN, β-LG, and S100 calcium-binding protein in goat’s milk and cow’s milk [26,36,37,38,39,40] (Figure 4). We also chose two protein spots with significant differences between the two breeds, to be identified by MALDI-TOF/TOF mass spectrometry.

**Figure 5:**
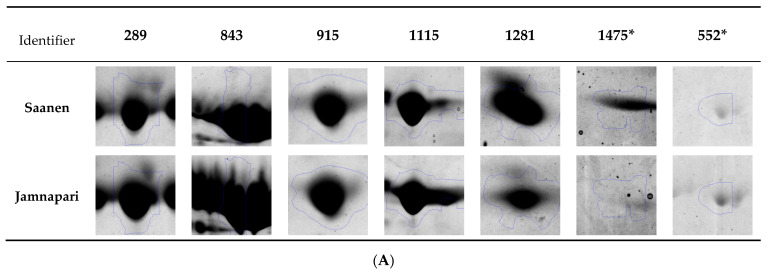

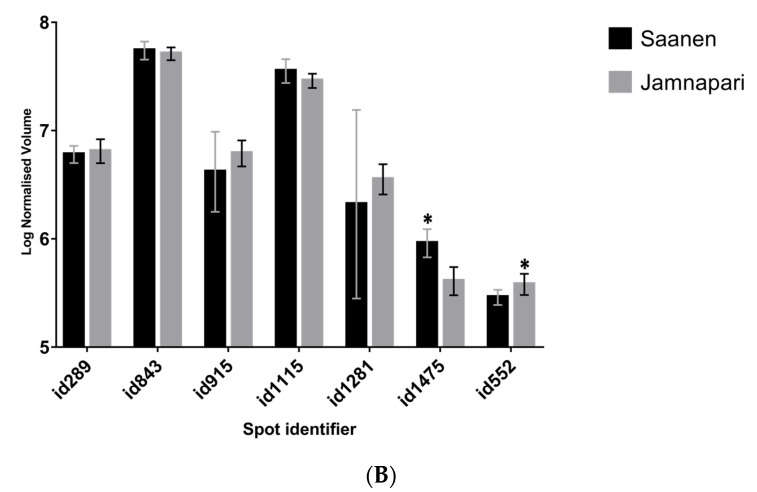
(**A**) Images of protein spots from 2DE gels of Saanen and Jamnapari goat’s milk extracted using method A that were chosen for identification by MALDI-TOF/TOF MS/MS. (**B**) Five spots from each breed were selected based on milk protein regions that were present in both breeds, with no significant differences in their levels, and two spots based on the significant differences in their abundance levels when analyzed by Progenesis SameSpot software. * Denotes protein spots with *p*-values < 0.05.

Five spots (protein ID 289, 843, 915, 1115, and 1281) were present in both breeds, with no significant differences in their levels, and two spots (protein id 1475 and 552) showed significant differences in their abundance levels when analyzed by Progenesis SameSpot software (Nonlinear Dynamic Ltd., Durham, NC, USA) (Figure 5A,B). Spot 1475 level was significantly higher in Saanen milk than Jamnapari, while spot 552 was significantly more abundant in Jamnapari milk than Saanen milk (Figure 5B).

All selected spots were positively identified via MALDI-TOF/TOF MS/MS. Identified protein spots were serum albumin, β-CN, β-LG, and actin, from the taxonomy of goat (*Capra hircus*), as well as sheep (*Ovis aries*) and cow (*Bos taurus*), which are known to be phylogenetically similar to goat species (Table 3).

## 3. Discussion

This study was conducted to determine the superior protein extraction procedure for the proteomic study of goat’s milk, which utilized gel-based protein separation before protein digestion and mass spectrometry analysis. The major goal of a protein extraction protocol is to solubilize protein of interest effectively and reproducibly; prevent any protein loss or aggregation during further analysis and post-extraction modification or degradation; and remove contaminant while yielding a detectable amount of proteins of interest. Currently, there is no standardized extraction method that has been established for the investigation of goat’s milk proteomes. Most of the protein extraction methods available from the literature typically focused on cow’s milk proteomic studies, and each of these methods was developed to suit the objectives of the respective studies. These include methods for the recovery of casein fraction by ultracentrifugation [42] and whey fraction by membrane filtration [43] or by the removal of casein fraction through acidification with acetic acid [44]. While extraction methods that focused on the fractionation of casein and whey proteins had been previously shown to enrich low abundant proteins in colostrum and mature cow’s milk [45], the laborious processes of casein and whey fractionation via acidification, ultrafiltration, and centrifugation may compromise the throughput. Thus, our study attempted to optimize the whole protein extraction method that is suited for the high-throughput 2DE-based proteomic analysis of goat’s milk. This can be achieved by comparing the efficacies of different extraction conditions in recovering and resolving the highest number of proteins from the fat-free goat’s milk.

Here, we are comparing three different extraction methods for total proteins in Saanen and Jamnapari goat’s milk. For method A, a skim milk sample diluted in a urea-based solubilization buffer was adapted from several studies utilizing 2DE gels [30,46]. However, we further modified the buffer by combining 7 M urea and 2 M thiourea as chaotropic agents. Previous studies had demonstrated that combining urea and thiourea in the extraction buffer resulted in an increase of the protein solubility and a minimization of the proteolysis in the extract [47,48,49,50]. In method A, instead of using sodium dodecyl sulphate (SDS), which is a harsh ionic detergent, a neutral charged zwitterion detergent such as 3-[(3-cholamidopropyl) dimethylammonio]- 1-propanesulfonate (CHAPS) was used, as it had been shown to perform better at hydrolyzing the protein–protein bonds, while maintaining the protein individual charge and native state [51,52]. The disulfide bond between proteins was reduced by the dithiothreitol (DTT) in the solubilizing buffer when incubated at 30 °C and was further stabilized by alkylation with iodoacetamide (IAA) at room temperature. For method B, we adopted a methanol/chloroform-based extraction method that was previously used for the cow’s milk protein extraction [29]. This method involved a phase-separation technique that took advantage of the solubility of lipid compound in chloroform and protein precipitation capability in methanol [53]. The addition of sodium chloride to the mixture of chloroform, methanol, and skim milk sample before the centrifugation step was to prevent any unnecessary binding of acidic lipid to the denatured lipid [54]. Sodium chloride was used to facilitate protein precipitation by saturating the hydrogen ion in water, thus promoting hydrophilic interaction between proteins to cause them to precipitate [55]. The buffer used in method B consisted of the elements necessary for protein solubilization, including urea as a chaotrope to disrupt the hydrogen binding, sodium dodecyl sulphate (SDS) as detergent to disaggregate the casein micelles, DTT to reduce the proteins disulfide bridges, and sodium chloride buffered in Tris-HCl, at pH 8.0, to increase the reduced milk protein stability. Method C was developed by a previous study as an effective allergen extraction method for processed food, in which a greener reductant, sodium sulfite, was used in place of the environmentally hazardous 2-mecarptoethanol (2-ME) [56]. As a reductant, sodium sulfite breaks down the disulfide bond by inhibiting free mercaptan groups and thus reducing the disulfide crosslinking [57]. In method C, skim milk was shaken overnight at room temperature with the solubilizing buffer that consists of Tris HCl, with the addition of Tween 20, SDS, and sodium sulfite at pH 7.4. Sodium sulfite had been proven for its capability to economically reduce disulfide bonds in minute amounts in other studies [58,59]. The previous studies established that sodium sulfite performed as well as 2-Mercaptoethanol in solubilizing milk, egg, wheat, peanuts, and buckwheat protein allergens for ELISA detection systems [56]. While the protein extracts had been evaluated on 1DE [56], the compatibility of the protocols for 2DE has not been conducted.

In comparison to method B and method C, method A was the simplest and fastest method, with the least protein loss during the extraction step. The total time required for method A was around 2.5 h. Method C, while requiring fewer steps compared to method B, still involved an overnight incubation of the samples in the extraction buffer. Whilst a previous study [29] reported method B recovering high concentration of cow’s milk protein extract, our study showed that method A recovered the highest goat’s milk protein yield and the highest number of protein spots. Upon evaluation of protein extract on 1DE, the protein bands produced from methods A and B extract were more intense compared to method C. Method B has shown comparable or slightly more intense protein bands on 1DE than method A, as supported by a study by Vincent at al. for cow’s milk proteome [29]. Two-dimensional electrophoretic analysis of each extract showed the dispersion of protein spots that were separated based on their isoelectric point and molecular weight. Acquired gel images analyzed using Progenesis SameSpot software (Nonlinear Dynamics, Durham, NC, USA) showed that the resolution of spots across 2DE gels produced from method A was the best compared to the other two methods. Similar extraction methods adopted by recent studies on Girgentana goat’s milk proteomics [36], which also utilized a combination of urea and thiourea in their extraction buffer, showed comparable protein spot resolutions to our finding here. The further addition of ampholytes (IPG buffer) in our extraction buffer for method A helped in improving the resolution of the protein spots on the 2DE gel and minimized streaking, compared to the findings by Di Gerlando et al. (2019) [36]. We managed to produce comparable qualities of 2DE gels that we attained from method A, in terms of total protein spots and resolution, to the previous studies on Girgentana goat’s milk [36] and Greek goat’s and sheep’s milk [60], despite using more economical and less labor-intensive 13 cm narrow pH range (pH 4–7) gels than the more expensive 18 cm wide pH range (pH 3–10) gels used in their studies. Narrow range strips (pH 4–7) used in our study also allowed us to separate proteins in the casein regions better than the previous studies on milk of Girgentana goats [36] and different breeds of Greek goats and sheep [60] that had used wider pH range strips (pH 3–10).

Comparatively, 2DE gels of extraction from method B showed poorer spot resolution with severe horizontal streaking. The horizontal streaking observed on gels from method B may be caused by poor protein solubilization during the isoelectric focusing. While the isoelectric focusing on all rehydrated IPG strips was conducted simultaneously, the presence of salt in the method B buffer caused interference in a certain region of the gels and under-focusing of IPG strips, thus resulting in horizontal streaking [61]. These complications led to poor resolution of spots across the gels and increased the possibilities of underestimating the total number of spots on the 2DE gels. Meanwhile, the 2DE gels attained from method C showed good protein spot resolutions, but the intensity and the total number of protein spots were the lowest. Method C produced well-separated proteins on a region previously reported as β-CN, serum albumin, β-LG, and α-LA [37]. The presence of 1% SDS, an anionic detergent in the method C buffer, was suspected to have caused the loss of proteins, as it can bind to a protein molecule and alter the structure and charges of the protein [54]. During isoelectric focusing, protein mobility was dictated by its net charge, as SDS denatures protein while imparting uniform negative charge on the protein, thus hindering the protein ability to stop at their respective isoelectric points [54]. In gels from method C, only proteins in the casein region were affected by the presence of SDS that resulted in lower protein spot resolution when compared to gels from method A.

Six protein spots from each breed (a total of 12 spots) from method A were further identified via MALDI-TOF/TOF MS/MS. Primarily, the spots of interest for identification were randomly selected based on previously reported protein regions [37,38,60], mainly CNs, β-LG, and serum albumin regions (Figure 4), with similar protein levels (Figure 5B). From the 2DE gel of Saanen milk samples, three spots (843S, 915S, and 1281S) were identified as β-CN, two spots (289S and 1475S) were identified as serum albumin, and one spot (1115S) was identified as β-LG. In the 2DE gel of Jamnapari milk samples, three spots (843J, 915J, and 1281J) were identified as β-CN; one spot (552J) was identified as Actin, cytoplasmic 1; one spot (289J) was identified as serum albumin; and one spot (1115J) identified as β-LG. As the five spots (289, 843, 915, 1281, and 1115) were taken from the same spots in the gels of different goat breeds, these 10 spots were identified as the same protein as their comparable breeds in the study. Identified spots were consistent to a previously reported protein on the same region, except for spot 1281, which was previously identified as S100 calcium-binding protein in cow’s milk [40]. As for the remaining two spots, one spot (1475S) was more significant on the 2DE gels of Saanen milk samples and had been identified as serum albumin, despite a previous report that identified mostly α-S1-casein on that region. Meanwhile, another spot (552S), which was more significant in Jamnapari milk samples, was identified as Actin, cytoplasmic 1, which coincides with a previously reported spot on the same region [60].

## 4. Materials and Methods

### 4.1. Sample Preparation

Goat’s milk samples from two different breeds, Saanen and Jamnapari, were collected from a commercial goat farm in Kluang, Johor, Malaysia. Six goats per breed, with a total of 12 samples, were milked manually in the early morning, on the farm site, and 100 mL of midstream milk samples was collected and used for the analysis. All animals were in their mid-lactation stage (3–6 months), bred on the same field condition, free of diseases and clinically healthy, similar to samples collected for previous studies by our group [35]. All milk samples were pooled according to their respective breeds, then aliquoted in sterile 2 mL tubes, and snap-frozen immediately in a dry-ice ethanol bath. Aliquoted milk sample was stored in −80 °C prior to use. Before extraction, milk samples were skimmed. Frozen milk samples were thawed at 4 °C, and 2 mL of milk was centrifuged at 4600 rpm for 30 min at 4 °C. The skim milk was recovered between the top fat layer and bottom pelleted cells, and the sample immediately underwent protein extraction process.

### 4.2. Protein Extraction

**Figure 6:**
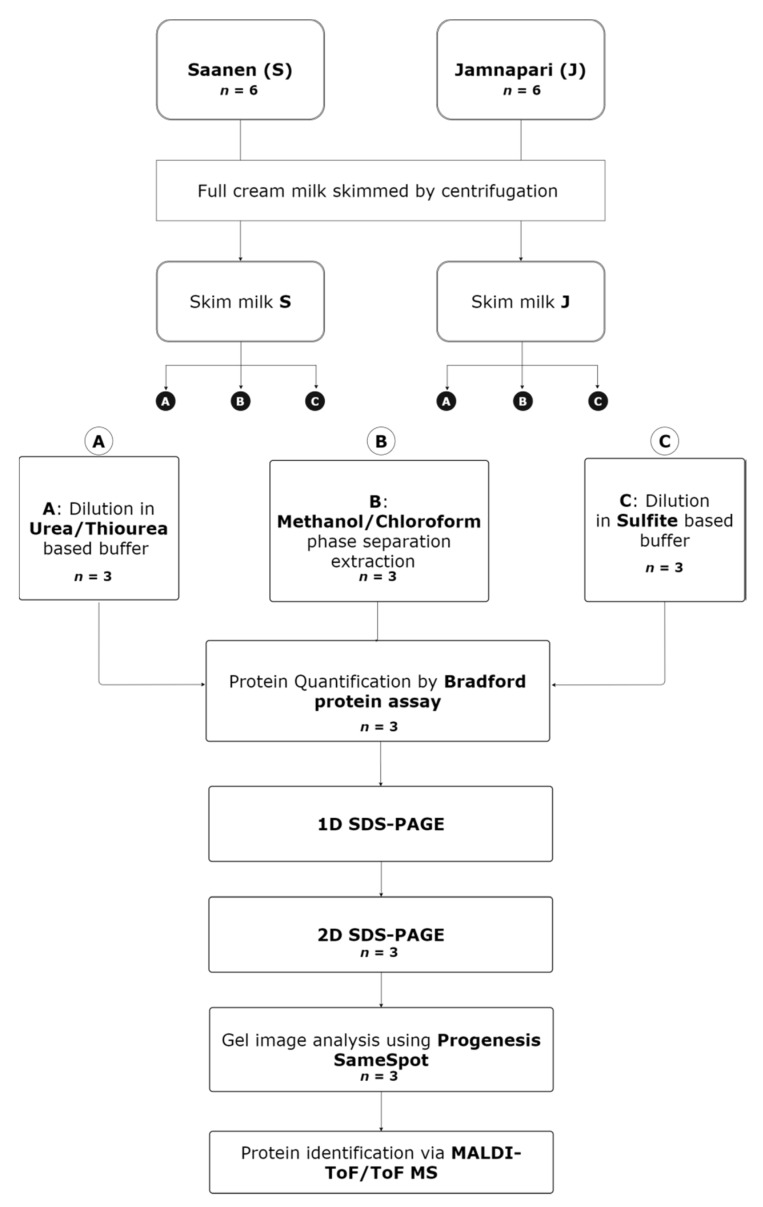
A workflow overview of the three extraction methods used in this study on milk proteins from Saanen and Jamnapari goats; ***n*** indicates the number of technical replicates used for each stage of the study.

Three different extraction methods were compared in this study on goat’s milk samples from two different breeds. The urea/thiourea-based method (method A), methanol/chloroform-based method (method B) and sulfite-based method (method C) were tested to ascertain the most fitting extraction method for the 2DE analysis of the goat’s milk samples. Milk samples of each breed were pooled from six biological replicates and divided into three technical replicates. Extraction methods A, B, and C, on each milk sample per breed, were performed in triplicates. An outline of the designed experiment for extraction methods is summarized in Figure 6.

#### 4.2.1. Method A (Urea/Thiourea)

Total milk protein extraction method adapted from [30,31,62] was performed, with some modification. Briefly, 0.5 mL of skim milk was added with Lysis Buffer (LB: 7M urea, 2M thiourea, 4% CHAPS, 2% IPG Buffer, and 40 mM dithiothreitol (DTT) in deionized water) at 1:1 ratio before the mixture was vortexed for 1 min. The mixture was then incubated for 1 h in a 30 °C water bath. Then, 1 M of Iodoacetamide (IAA) solution was added into the mixture, to reach a final concentration of 20 mM, before being incubated in the dark, at room temperature, for 1 h. The mixture was then centrifuged at 13,000 rpm for 5 min, at room temperature, before being kept at −80 °C prior to further analysis.

#### 4.2.2. Method B (Methanol/Chloroform)

A phase-separation extraction method using methanol and chloroform was adapted from Vincent, et al. (2016) and Taylor and Savage (2006) [29,53]. Briefly, 0.5 mL of skim milk was aliquoted into 50 mL tubes before subsequently adding 7.5 mL of chloroform and methanol at a ratio of 1:2, 5 mL chloroform, and 2 mL of NaCl (1:10 (w:v)) and mixing by vortexing, after each addition, for 1 min. The mixture was further centrifuged at 5100 rpm, at room temperature, for 30 min, to form the triphasic solution. The wet interphase was collected after carefully removing the upper and lower phases and vacuum-dried using a ScanSpeed MiniVac Evaporator (Saur, Reutlingen, Germany) at 1500 rpm at 30 °C for 60 min. Dried interphase was then resuspended in 0.5 mL of Solubilizing buffer (6 M urea, 10 mM DTT, 75 mM NaCl, 10 mM Tris-HCl pH 8.0, and 0.05% SDS in deionized water) and incubated at 4 °C overnight. The interphase was then vortexed for 30 min, added with 1 M of IAA solution to make a final 20 mM concentration, and further incubated in the dark, at room temperature, for an hour. Protein extracts were then kept at −80 °C until further analysis.

#### 4.2.3. Method C (Sodium Sulfite)

The sulfite-based extraction method was adapted and modified from Ito et al. (2016) [56]. Briefly, 0.5 mL of skim milk was aliquoted into 2 mL tubes before adding the same amount of buffer containing 7% (*w*/*v*) sodium sulfite, 120 mM Tris-HCl (pH 7.4), 1% (*w*/*v*) SDS, and 0.05% (*v*/*v*) Tween 20. The sample was shaken on a shaker, at 110 rpm, overnight, at room temperature. The mixture was later centrifuged at 3000× *g* for 20 min. Protein extract was obtained after filtering the supernatant. Protein extract was stored at −80 °C until further analysis.

### 4.3. Protein Quantification using Bradford Assay

Bradford protein assay (Biorad Laboratories, Hercules, CA, USA) was performed on each protein extract and skim milk sample, to determine their respective protein concentration. Seven dilutions of Bovine Serum Albumin protein standard (Biorad Laboratories, Hercules, CA, USA), ranging from 0.125 to 2 mg/mL, were prepared, to establish a standard curve. Then, 10 µL of unidentified sample and protein standard solution was added into each well in a 96-well plate, followed by 200 µL of Bradford reagent, before 5 min incubation at room temperature. Absorbance of each well was measured by using a Biotek Synergy 2 microplate reader (Biotek Instrument Inc., Winooski, VT, USA) at 595 nm. The standard curves were extrapolated as a simple linear regression model with R squared to be at least 0.99 and equation y = mx + c, where m is the slope and c is the crossing point at the *y*-axis. The protein concentration (mg/mL) correspondent to the absorbance reading at 595 nm obtained from each sample dilution was calculated by using this equation. Protein concentration determination was carried out in triplicate.

### 4.4. One-Dimensional Polyacrylamide Gel Electrophoresis

For each protein-extract quality evaluation, one-dimensional sodium dodecyl sulphate polyacrylamide gel electrophoresis (1D SDS-PAGE) was carried out according to Laemmli [63]. Skim milk and protein extract amounting to 20 µg of protein was diluted 1:1 with sample loading solution (0.5M Tris-HCl, pH 6.8, 10% SDS, Glycerol, and Bromophenol Blue), to reach a final volume of 10 µL before being heated at 95 °C for 5 min. The protein samples and skim milk were then run through 5% stacking gel, followed by 12% resolving gel at 90 V, using a Mini-Protean system (Biorad Laboratories, Hercules, CA, USA). Biorad Precision Plus Protein Dual Color Standards (Biorad Laboratories, Hercules, CA, USA) were loaded in the first lane of each gel, to estimate the molecular weight of proteins. The gels were stained by using Coomassie Brilliant Blue (CBB) staining protocol. Gels were stained with CBB staining solution, on a shaker, for 6 h, at room temperature, before being destained with destaining solution (40% methanol and 7% acetic acid in H_2_O) for 40 min.

### 4.5. D Polyacrylamide Gel Electrophoresis

The protein extract of each breed attained from each method was then run on the 2DE, in triplicate. Rehydration of the IPG strips (GE Healthcare, Chicago, IL, USA) was performed overnight, with the presence of 400 µg of protein samples. Isoelectric focusing (IEF) was performed on 13 cm pH 4–7 IPG strip (GE Healthcare, Chicago, IL, USA), using the Ettan IPGphor 2 IEF system (GE Healthcare, Chicago, IL, USA) at 500 V/4 h, 1000 V/1 h, 5000 V/1 h, 8000 V/1 h, and lastly at 29,000 V/1 h. The strips that had been equilibrated were then placed on polyacrylamide gels, and the electrophoresis was run on Ruby SE 600 electrophoresis system (GE Healthcare, Chicago, IL, USA) for 15 min at 20 mA/gel and 4 h at 40 mA/gel. After the run was completed, the 2DE gels were further fixed in gel-fixing solution (40% methanol and 10% acetic acid in H_2_O) overnight before being stained with CBB staining solution.

### 4.6. Image Acquisition and Statistical Analysis

Visualization of 1D and 2DE gels was performed by using Biorad GS800 calibrated densitometer (Biorad Laboratories, Hercules, CA, USA) with a 600 dpi resolution and 32-bit pixel depth. Acquired images of 2DE gels were subjected to automated single stain analysis, using Progenesis SameSpot software version 3.1v (Nonlinear Dynamic Ltd., Durham, NC, USA), in which the normalized volume was computed and each spot was assessed for its differential abundance, using ANOVA test. A total number of spots for each condition was counted after examining the presence of each spot in all gels. Protein spots with significant difference (*p* < 0.05) and >1.5-fold changes were tagged. We further performed the power analysis to confirm significant expression changes between the replicates and the number of replicates required to achieve the targeted 80% of power threshold. Six proteins spots were then selected from gels of each breed, for protein identification.

### 4.7. In Gel Protein Digestion

Desired spots from 2DE gels were excised, destained in washing solution (50% Acetonitrile in 100 mM ammonium bicarbonate, (NH_4_) HCO_3_), and then reduced in reduction solution (100 mM DTT in 100 mM (NH_4_) HCO_3_) for 30 min, in a 60 °C water bath. The reduced samples were then added with alkylation solution (55 mM IAA in 100 mM (NH_4_) HCO_3_) and incubated in the dark for 20 min. After washing and incubating the samples in 100% acetonitrile (ACN) for 15 min, the samples were dried, using the ScanSpeed MiniVac Evaporator (Saur, Reutlingen, Germany) for 1 h. Then, the samples were digested by adding 7 µg/µL trypsin solution and incubating at 30 °C overnight. The digested peptides were then mixed and spun with 100% can; the supernatant was collected and then dried using ScanSpeed MiniVac Evaporator (Saur, Reutlingen, Germany) for 1 h. Dried peptides were kept at −80 °C until further mass spectrometric analysis.

### 4.8. Protein Identification by MALDI-TOF/TOF Mass Spectrometry

Dried peptides were reconstituted in TA30 solution (0.1% TFA in 30% ACN), desalted using Zip-Tip C18 (Millipore, Bedford, MA, USA), and analyzed on an Ultraflextreme MALDI-TOF/TOF mass spectrometer (Bruker, Bremen, Germany). The spectra were then analyzed by using MASCOT search version 3.5 (Matrix Science, Boston, MA, USA) against taxonomy mammal entries against Swissprot database, to identify the proteins of interest. The parameters used for database searches were as follows: The enzyme was set as trypsin with one missed cleavage allowed, with a fragment mass tolerance of 1.0 Da, a peptide mass tolerance of 200 ppm, methionine oxidation as a variable modification, carbamidomethylation as a fixed modification, and mass values as monoisotopic.

## 5. Conclusions

In conclusion, three different total protein extraction methods conducted on goat’s milk samples from two different goat’s breeds, Saanen and Jamnapari, were evaluated. Milk protein extract from each method was analyzed and compared, using Bradford Protein Assay and 1D and 2D gel electrophoresis, and the proteins were identified by MALDI-TOF/TOF mass spectrometry. Method A (urea/thiourea) yielded the most concentrated protein extract and resolved most spots on 2DE gels with the superior spot resolution and the least streaking, hence making it our method of choice for our subsequent allergenomic study on goat’s milk. Although method B showed a good 1DE profile and recovered a decent amount of protein spots, it produced 2DE gel with severe streaking and the worst spot resolution. As for method C, despite being efficient for food allergen extraction method for ELISA, it is not suitable for an optimum gel-based proteomic analysis that utilized 2DE. While 2DE proteomics involves labor-intensive procedures and may result in a low dynamic range of proteins, these limitations can be overcome by optimizing the protein extraction methods and using narrow-range pH strips for higher resolution of separated proteins on 2D format, as has been demonstrated here. Findings from this study can be applied in our future allergenomic study on the characterization and quantification of major milk allergens from different goat breeds, as well as the IgE epitope mapping of goat’s milk allergens. The application of this approach will contribute toward the diagnosis of goat’s milk allergy and improvement of the safety assessment of goat’s milk from different breeds, to be used as milk alternatives for CMPA patients.

## Figures and Tables

**Table 1:** The concentration of proteins extracted from milk of Saanen and Jamnapari goats, using different extraction methods.

Extraction Method	Protein Concentration (mg/mL)	
	Saanen	Jamnapari
Method A (Urea/Thiourea)	120.54 ± 22.26	134.62 ± 18.76
Method B (Methanol/Chloroform)	94.29 ± 13.87	98.52 ± 3.15
Method C (Sulfite)	75.99 ± 6.95	92.88 ± 8.74

**Table 2:** Total number of detectable spots from 2DE gels of Saanen and Jamnapari goat’s milk extracted by using three different extraction methods.

Extraction Method	Number of Spots	
	Saanen	Jamnapari
Method A	199 ± 16.1	267 ± 10.6
Method B	192 ± 9.8	219 ± 13.6
Method C	192 ± 10.6	204 ± 26.5

**Table 3:** Characterization of protein spots by MALDI-TOF/TOF MS/MS.

Spot	Protein	Taxonomy	Accession no.	Score	Nominal Mass	Calculated pI	Matches	Coverage/%	Peptide Sequence	Function
**289S**	Serum albumin	*Ovis aries*	P14639	232	71,139	5.8	5	11	R.RHPYFYAPELLYYANK.Y K.DVFLGSFLYEYSR.R R.RHPEYAVSVLLR.L K.HGEYGFQNALIVR.Y R.MPCTEDYLSLILNR.L	Regulator of the blood osmotic pressure and major zinc transporter in plasma
**843S**	Beta-casein	*Capra hircus*	P33048	74	24,906	5.26	2	8	R.DMPIQAFLLYQEPVLGPVR.G R.DMPIQAFLLYQEPVLGPVR.G	Determinant of the casein micelles surface properties
**915S**	Beta-casein	*Capra hircus*	P33048	79	24,906	5.26	3	17	K.YPVEPFTESQSLTLTDVEK.L R.DMPIQAFLLYQEPVLGPVR.G R.DMPIQAFLLYQEPVLGPVR.G	Determinant of the casein micelles surface properties
**1115S**	Beta-lactoglobulin	*Capra hircus*	P02756	68	20,362	5.5	3	27	K.VAGTWYSLAMAASDISLLDAQSAPLR.V K.VAGTWYSLAMAASDISLLDAQSAPLR.V K.YLLFCMENSAEPEQSLACQCLVR.T	Major component of whey, probably involved in the transport of retinol
**1281S**	Beta-casein	*Capra hircus*	P33048	83	24,906	5.26	1	8	R.DMPIQAFLLYQEPVLGPVR.G	Determinant of the casein micelles surface properties
**1475S**	Serum albumin	*Ovis aries*	P14639	95	71,139	5.8	3	7	R.RHPYFYAPELLYYANK.Y K.DVFLGSFLYEYSR.R R.MPCTEDYLSLILNR.L	Regulator of the blood osmotic pressure and major zinc transporter in plasma
**289J**	Serum albumin	*Ovis aries*	P14639	204	71,139	5.8	4	9	R.RHPYFYAPELLYYANK.Y K.DVFLGSFLYEYSR.R K.HGEYGFQNALIVR.Y R.MPCTEDYLSLILNR.L	Regulator of the blood osmotic pressure and major zinc transporter in plasma
**552J**	Actin, cytoplasmic 1	*Bos taurus*	P60712	67	42,052	5.29	2	7	K.IWHHTFYNELR.V K.SYELPDGQVITIGNER.F	Highly conserved proteins that are involved in different types of cell motility and are abundantly expressed in eukaryotic cells.
**843J**	Beta-casein	*Capra hircus*	P33048	64	24,906	5.26	2	8	R.DMPIQAFLLYQEPVLGPVR.G R.DMPIQAFLLYQEPVLGPVR.G	Determinant of the casein micelles surface properties
**915J**	Beta-casein	*Capra hircus*	P33048	68	24,906	5.26	2	8	R.DMPIQAFLLYQEPVLGPVR.G R.DMPIQAFLLYQEPVLGPVR.G	Determinant of the casein micelles surface properties
**1115J**	Beta-lactoglobulin	*Capra hircus*	P02756	77	20,362	5.5	2	14	K.VAGTWYSLAMAASDISLLDAQSAPLR.V K.VAGTWYSLAMAASDISLLDAQSAPLR.V	Major component of whey, probably involved in the transport of retinol
**1281J**	Beta-casein	*Capra hircus*	P33048	45	24,906	5.26	1	8	R.DMPIQAFLLYQEPVLGPVR.G	Determinant of the casein micelles surface properties
